# Clinical features and prognostic factors of *Chlamydia psittaci* pneumonia: a retrospective study

**DOI:** 10.3389/fmed.2026.1804156

**Published:** 2026-04-02

**Authors:** Yanyan Li, Hongyi Zhu, Zijie Zhan, Ge Li, Quan Zhou, Chao Zheng, Fan Huang

**Affiliations:** 1 Department of Respiratory and Critical Diseases, The Affiliated Changde Hospital of Xiangya School of Medicine, Central South University, Changde, Hunan, China; 2 Department of Science and Education Section, The Affiliated Changde Hospital of Xiangya School of Medicine, Central South University, Changde, Hunan, China; 3 Department of Radiology, The Affiliated Changde Hospital of Xiangya School of Medicine, Central South University, Changde, Hunan, China; 4 Department of Quality Control Department, The Affiliated Changde Hospital of Xiangya School of Medicine, Central South University, Changde, Hunan, China

**Keywords:** *Chlamydia psittaci*, clinical features, next-generation sequencing (NGS), prognosis, psittacosis pneumonia

## Abstract

**Background:**

*Chlamydia psittaci* pneumonia (CPP) is frequently misdiagnosed and can progress to severe illness. A deeper understanding of its clinical and imaging features is crucial for early detection and effective treatment.

**Methods:**

This retrospective study analyzed 74 patients diagnosed with CPP via metagenomic (mNGS) and targeted next-generation sequencing (tNGS) between January 2022 and September 2025. Patients were categorized into severe (*n* = 21) and non-severe (*n* = 53) groups based on established criteria for severe community-acquired pneumonia. Data on demographics, clinical manifestations, laboratory findings, and imaging characteristics were collected and compared.

**Results:**

The cohort had a median age of 60 years, with a male predominance (62.2%). A history of poultry/bird exposure was reported by 87.8% of participants. Common symptoms included fever (94.6%), cough (63.5%), and fatigue (29.7%), with no significant differences between groups. Hospitalization was significantly longer in the severe group (12.95 ± 6.08 days) than in the non-severe group (8.13 ± 3.30 days) (*p* < 0.001). Chest CT revealed consolidation and ground-glass opacities in all patients. Pleural effusion was significantly more common in the severe group (76.2% vs. 45.3%, *p* = 0.016), as was bilateral lung involvement (52.4% vs. 22.6%, *p* = 0.013). Multivariate analysis identified elevated D-dimer (OR = 2.737, *p* = 0.007) and reduced lymphocyte percentage (L%) (OR = 0.813, *p* = 0.026) as independent predictors of severe disease. ROC curve analysis showed an AUC of 0.765 for D-dimer and 0.739 for L% reduction. Following tetracycline or quinolone therapy, 94.6% of patients recovered, with an overall mortality rate of 5.4%.

**Conclusion:**

Severe CPP is associated with prolonged hospitalization, bilateral pulmonary infiltrates, and pleural effusion. D-dimer and lymphocyte percentage are valuable prognostic indicators for disease severity. Early targeted antibiotic therapy is effective, but timely respiratory support is critical for severe cases.

## Introduction

*Chlamydia psittaci* is a Gram-negative, obligate intracellular bacterium that predominantly infects avian species, poultry, and humans (1). *Chlamydia psittaci* pneumonia (CPP), a zoonotic disease induced by *C. psittaci*, has its root in avian species, and humans generally acquire the infection through inhalation of contaminated aerosols (2). Human-to-human transmission has also been rarely reported (3). As a type of community-acquired pneumonia, the comprehensive spectrum of its clinical features remains to be fully clarified. Prior research has reported a high incidence rate of severe pneumonia, reaching up to 7.5% (4), highlighting the crucial significance of early identification and timely treatment.

The disease predominantly manifests as pulmonary symptoms and is frequently accompanied by systemic manifestations, including high fever, cough, fatigue, myalgia, and dizziness (5). Fortunately, almost all patients exhibit a favorable response to quinolones and tetracyclines, leading to an excellent recovery rate (6). Notwithstanding this efficacious treatment, clinical diagnosis persists as a formidable challenge owing to the non-specific characteristics of its manifestation and the constraints of traditional diagnostic approaches. The emergence of next-generation sequencing (NGS) technology has resulted in a substantial upsurge in the reported cases of CPP (7). Previous studies have indicated that metagenomic NGS (mNGS) serves as a crucial novel diagnostic instrument, particularly for the accurate identification of rare pathogens (8). Analogously, targeted NGS (tNGS) facilitates early and precise diagnosis, presenting enhanced cost-effectiveness (9).

Nevertheless, the factors that can predict the progression of the disease to severe forms remain ambiguous, which hinders the formulation of early intervention strategies. This study conducted a retrospective analysis of patients diagnosed with CPP. Through the comparison of diverse clinical, laboratory, and imaging indicators between severe and non-severe cases, this research intends to offer valuable clinical perspectives for the early diagnosis and management of psittacosis, thereby ultimately contributing to the improvement of outcomes for critically ill patients.

## Materials and methods

**Figure 1:**
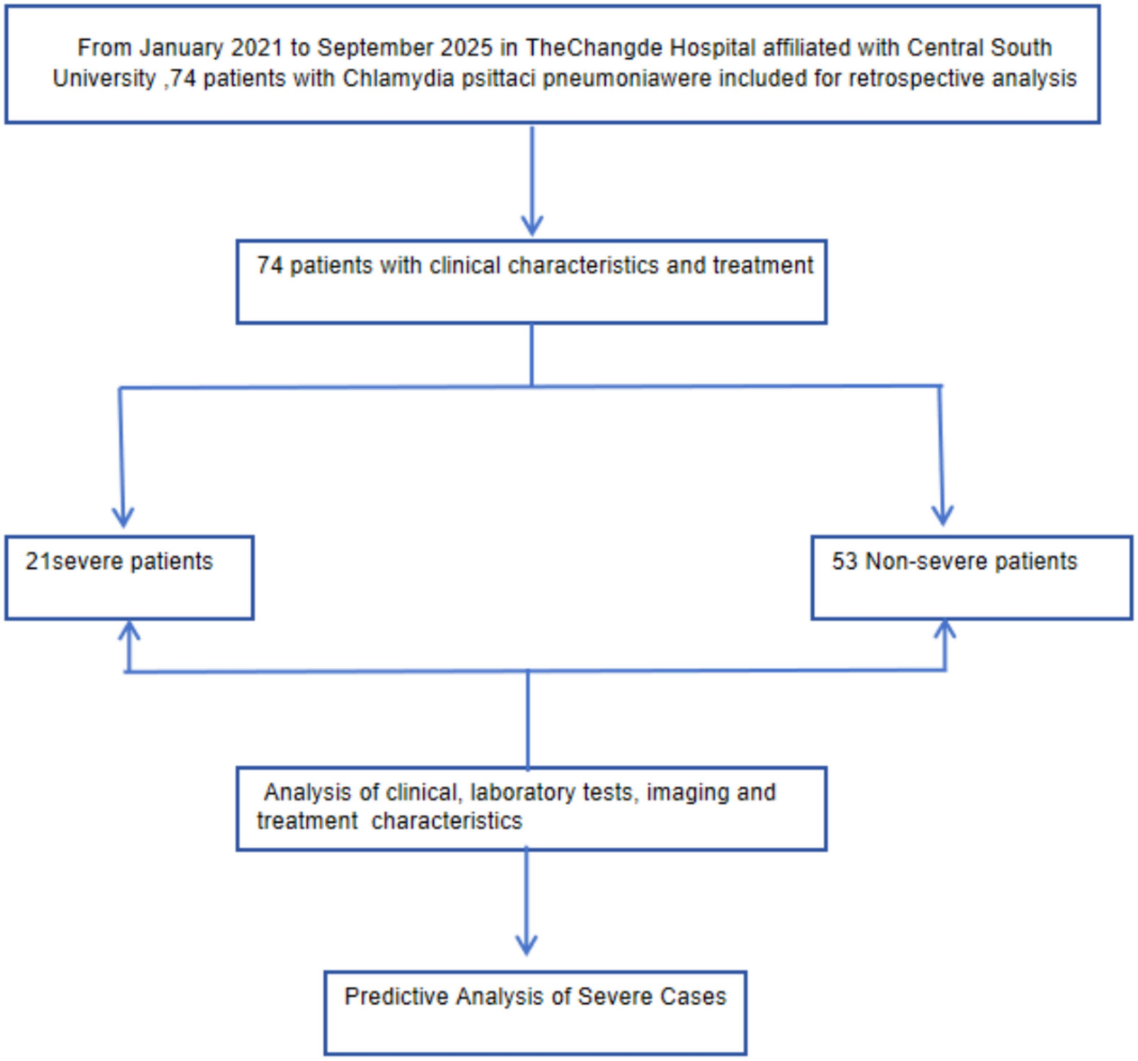
Research route for this study design.

Between January 2022 and September 2025, a total of 74 cases of psittacosis were confirmed at the Changde Hospital affiliated with Central South University. All cases were diagnosed through both mNGS and tNGS. According to the Infectious Diseases Society of America/American Thoracic Society consensus guidelines for severe community-acquired pneumonia (10), 21 patients were categorized as having severe pneumonia, while 53 were classified as having non-severe pneumonia (Figure 1).

### Data collection

This retrospective cohort study, carried out at The First People’s Hospital of Changde City from January 2022 to December 2025, incorporated patients diagnosed with *C. psittaci* infection through mNGS or tNGS of alveolar lavage fluid, sputum, or peripheral blood. Subsequently, these patients were stratified into severe and non-severe groups in accordance with the established diagnostic criteria for severe community-acquired pneumonia (either necessitating mechanical ventilation/septic shock or fulfilling ≥3 secondary criteria). This study amassed comprehensive data encompassing demographics, comorbid conditions, clinical manifestations, laboratory test results (such as complete blood count (CBC), inflammatory biomarkers, and biochemical profiles), chest computed tomography (CT) findings, treatment regimens, and clinical outcomes. Complications occurring during hospitalization, including respiratory failure, sepsis, acute respiratory distress syndrome (ARDS), acute kidney injury, cardiac failure, disseminated intravascular coagulation (DIC), and anemia, were assessed using internationally recognized standardized criteria. Detailed definitions for each complication are provided in the Supplementary Appendix. The primary aims were to discern independent predictors for severe pneumonia through multivariate logistic regression analysis and to assess the diagnostic performance of predictive indicators via receiver operating characteristic (ROC) curve analysis (including area under the curve AUC, sensitivity, and specificity). The secondary outcomes centered on comparing clinical characteristics, laboratory and imaging findings, and treatment efficacy between the two groups.

This study strictly complies with the principles of the Declaration of Helsinki and has been approved by the Medical Ethics Committee of Changde Hospital, Xiangya School of Medicine, Central South University, China (Approval No. 2026-042-01). Informed consent was waived due to the retrospective study design.

### NGS and analysis

This study utilized the DNBSEQ sequencing platform to conduct both mNGS and tNGS, As described in previous studies (11). The technical principle of mNGS entails the comprehensive examination of all nucleic acids (DNA and RNA) within a sample. By leveraging high-throughput sequencing technology, it randomly sequences the genomes of microbial communities in the sample to generate metagenomic data, which is then compared with known microbial databases to identify the specific pathogenic microorganisms. The criteria for interpreting positive mNGS results are as follows: for intracellular bacteria (e.g., *C. psittaci*, Mycobacterium spp.) and parasites, which have a low risk of contamination and pose difficulties in DNA extraction, a detection threshold of ≧1.2 reads per million (RPM) is regarded as positive; for opportunistic pathogens, fungi, and viruses, a sequence count more than five times higher than that of the control group is considered positive (12, 13). Conversely, the principle of tNGS is based on the targeted enrichment of specific pathogen nucleic acid sequences through methods such as highly multiplexed PCR or probe hybridization capture. Subsequently, high-throughput sequencing and database comparison are carried out to achieve the precise identification of common clinical pathogens (including bacteria, fungi, viruses, and parasites) and associated drug resistance genes. The interpretation criteria for tNGS are: specific sequences with ≥ 100 counts and multi-target coverage are directly classified as positive; sequences with counts ranging from 10 to 100 necessitate further clinical or methodological validation (14).

Regarding sample collection, typically 3–4 mL of bronchoalveolar lavage fluid or sputum is submitted for sequencing; when these samples are unavailable, peripheral blood is used for testing (15).

### Statistical analysis

All statistical analyses were carried out using SPSS version 26.0 (IBM Corp., Armonk, NY, USA), with a two-sided *p*-value <less than 0.05 regarded as statistically significant. Continuous variables conforming to a normal distribution were compared via the independent samples *t*-test and presented as the mean ± standard deviation. Meanwhile, non-normally distributed continuous variables were analyzed by the Mann–Whitney *U* test and expressed as the median (interquartile range, IQR). For comparisons among three or more groups, the Kruskal–Wallis test was employed. Categorical variables were analyzed using the Chi-square test or Fisher’s exact test, as applicable. Multivariable logistic regression analysis was conducted to identify independent predictors, and the predictive performance of relevant indicators for severe psittacosis was assessed using receiver operating characteristic (ROC) curve analysis.

## Results

### Demographic characteristics and clinical manifestations

Among the 74 enrolled patients, 87.8% reported a definite history of exposure to birds or poultry. The cohort was composed of 46 males (62.2%) and 28 females (37.8%). In the non-severe group (*n* = 53), 34 patients were male (64.2%) and 19 were female (35.8%), whereas the severe group (*n* = 21) included 12 males (57.1%) and 9 females (42.9%). No significant disparity in gender distribution was detected between the two groups (*p* = 0.575).

The overall median age was 60 years (IQR: 54.75–72.25). The median age in the non-severe group was 59 years (IQR: 54.0–72.5), and in the severe group, it was 63 years (IQR: 57.0–72.5), with no statistically significant difference (*p* = 0.197). The overall smoking rate was 33.8% (*n* = 25), with rates of 34.0% (*n* = 18) in the non-severe group and 33.3% (*n* = 7) in the severe group. This difference was not statistically significant (*p* = 0.959). The mean BMI was 23.5 ± 3.1 kg/m^2^ overall, measuring 23.9 ± 3.0 kg/m^2^ in the non-severe group and 22.4 ± 3.1 kg/m^2^ in the severe group, with no significant inter-group difference (*p* = 0.090).

The median hospital stay for the entire cohort was 9.50 ± 4.76 days. Nevertheless, the severe group had a significantly longer hospitalization duration (12.95 ± 6.08 days) compared to the non-severe group (8.13 ± 3.30 days) (*p* < 0.001). The median time from exposure to symptom onset was 7 days, with IQRs of 4–10 days in the non-severe group and 5–10 days in the severe group, suggesting no significant difference between the groups (*p* = 0.635).

Fever was observed in 94.6% (70/74) of patients, specifically 94.3% (50/53) in the non-severe group and 95.2% (20/21) in the severe group. This indicates a high incidence rate, and there was no significant difference between the groups (*p* = 1.000). Cough was reported in 63.5% (47/74) of patients, with similar proportions in the non-severe (64.2%, 34/53) and severe (61.9%, 13/21) groups (*p* = 0.856). Sputum production was reported by 47.3% (35/74) of patients, with a marginally higher incidence in the non-severe group (50.9%, 27/53) compared to the severe group (38.1%, 8/21). However, this difference was not statistically significant (*p* = 0.318). Dyspnea was present in 24.3% (18/74) of patients, with a markedly higher incidence in the severe group (38.1% vs. 18.9%). Nevertheless, this difference approached but did not attain statistical significance (*p* = 0.082).

Symptoms of the digestive system were less prevalent. Vomiting occurred in 13.5% (10/74) of patients, abdominal pain in 2.7% (2/74), and diarrhea in 5.4% (4/74). There were no significant differences between the severe and non-severe groups (*p* = 0.715, *p* = 0.490, and *p* = 0.572, respectively). Systemic symptoms included headache (21.6%, 16/74), dizziness (31.1%, 23/74), fatigue (29.7%, 22/74), and myalgia (20.3%, 15/74), with comparable proportions in the two groups (all *p*-values > 0.05). Loss of appetite was reported by 17.6% (13/74) of all patients, with a higher proportion in the severe group (28.6% vs. 13.2% in the non-severe group). Yet, this difference was not statistically significant (*p* = 0.173).

Regarding vital signs, a significant difference in heart rate was observed between the groups: the non-severe group demonstrated a mean value of 100.0 ± 9.4 bpm, whereas the severe group presented with a significantly elevated mean of 113.9 ± 17.4 bpm (*p* = 0.002). Similarly, respiratory rates exhibited a marked discrepancy, with non-severe patients averaging 21.8 ± 1.7 bpm compared to 26.4 ± 6.1 bpm among severe cases, a difference that also reached statistical significance (*p* = 0.003) (Table 1).

**Table 1:** Clinical manifestations of patients with CPP.

Characteristic	CPP (*n* = 74)	Non severe CPP (*n* = 53)	Severe CPP (*n* = 21)	*p*-value
Demographics				
Male	46 (62.2%)	34 (64.2%)	12 (57.1%)	0.575
Age, median (range, years)	60 (54.75, 72.25)	59 (54,72.5)	63 (57,72.5)	0.197
Length of stay (days)	9.50 ± 4.76	8.13 ± 3.30	12.95 ± 6.08	<0.001
Initial onset (days)	7 (4,10)	7 (4,10)	7 (5,10)	0.635
Smoking history	25 (33.8%)	18 (34.0%)	7 (33.3%)	0.959
BMI	23.5 ± 3.1	23.9 ± 3.0	22.4 ± 3.1	0.090
Clinical manifestations, *n*				
Fever	70 (94.6%)	50 (94.3%)	20 (95.2%)	1.000
Cough	47 (63.5%)	34 (64.2%)	13 (61.9%)	0.856
Expectoration	35 (47.3%)	27 (50.9%)	8 (38.1%)	0.318
Dyspnea	18 (24.3%)	10 (18.9%)	8 (38.1%)	0.082
Chest tightness	5 (6.8%)	3 (5.7%)	2 (9.5%)	0.618
Headache	16 (21.6%)	13 (24.5%)	3 (14.3%)	0.532
Dizziness	23 (31.1%)	16 (30.2%)	7 (33.3%)	0.792
Emesis	10 (13.5%)	8 (15.1%)	2 (9.5%)	0.715
Diarrhea	4 (5.4%)	4 (7.5%)	0 (0)	0.572
Abdominal pain	2 (2.7%)	1 (1.9%)	1 (4.8%)	0.490
Anorexia	13 (17.6%)	7 (13.2%)	6 (28.6%)	0.173
Fatigue	22 (29.7%)	13 (24.5%)	9 (42.9%)	0.120
Myalgia	15 (20.3%)	12 (22.6%)	3 (14.3%)	0.532
Vital signs, bpm				
Heart rate	103.9 ± 13.6	100.0 ± 9.4	113.9 ± 17.4	0.002
Respiratory rate	23.1 ± 13.6	21.8 ± 1.7	26.4 ± 6.1	0.003

BMI, body mass index.

### Complications and comorbidities

The severe group demonstrated notably higher complication rates in comparison to the non-severe group (*p* < 0.05). These complications encompassed respiratory failure (76.2% vs. 7.5%, *p* < 0.001), sepsis (23.8% vs. 1.9%, *p* = 0.006), renal insufficiency (42.9% vs. 7.5%, *p* < 0.001), metabolic acidosis (23.8% vs. 3.8%, *p* = 0.017), cardiac insufficiency (23.8% vs. 5.7%, *p* = 0.037), ARDS (14.3% vs. 0%, *p* = 0.021), and anemia (42.9% vs. 18.9%, *p* = 0.033). Other complications, such as DIC, acute pancreatitis, and electrolyte disturbances, occurred at low frequencies, and there were no significant inter-group differences. Nevertheless, their potential risks in severe cases necessitate vigilant monitoring.

In terms of comorbidities, common chronic conditions, including hypertension (33.8%), heart disease (9.5%), and diabetes (17.6%), were uniformly distributed between the two groups (all *p* > 0.05). Neurological disorders were more prevalent in the severe group (14.3% vs. 1.9%, *p* = 0.066), approaching statistical significance, which underscores the requirement for heightened attention to neurological involvement in severe psittacosis cases (Table 2).

**Table 2:** Complication of patients with CPP.

Characteristics	CPP (*n* = 74)	Non severe CPP (*n* = 53)	Severe CPP (*n* = 21)	*p-*value
Complication				
Respiratory failure	20 (27.0%)	4 (7.5%)	16 (76.2%)	<0.001
ARDS	3 (4.1%)	0 (0)	3 (14.3%)	0.021
DIC	1 (1.4%)	0 (0)	1 (4.8%)	0.284
Sepsis	6 (8.1%)	1 (1.9%)	5 (23.8%)	0.006
Metabolic acidosis	7 (9.5%)	2 (3.8%)	5 (23.8)	0.017
Anemia	19 (25.7)	10 (18.9%)	9 (42.9%)	0.033
Acute pancreatitis	1 (1.4%)	0 (0)	1 (4.8%)	0.284
Hepatic insufficiency	40 (54.1%)	27 (50.9%)	13 (61.9%)	0.394
Cardiac insufficiency	8 (10.8%)	3 (5.7%)	5 (23.8%)	0.037
Renal insufficiency	13 (17.6%)	4 (7.5%)	9 (42.9%)	<0.001
Electrolyte disturbance	35 (47.3%)	23 (43.4%)	12 (57.1%)	0.286
Comorbidities				
COPD	1 (1.4%)	0 (0)	1 (4.8%)	0.284
Tuberculosis	3 (4.1%)	3 (5.7%)	0 (0)	0.554
Hypertension	25 (33.8%)	16 (30.2%)	9 (42.9%)	0.299
heart disease	7 (9.5%)	6 (11.3%)	1 (4.8%)	0.665
Diabetes	13 (17.6%)	10 (18.9%)	3 (14.3%)	0.747
Tumor	4 (5.4%)	2 (3.8%)	2 (9.5%)	0.318
Hepatic disease	1 (1.4%)	0 (0)	1 (4.8%)	0.284
Operative history	5 (6.8%)	4 (7.5%)	1 (4.8%)	1.000
Neurological disorders	4 (5.4%)	1 (1.9%)	3 (14.3%)	0.066

ARDS, acute respiratory distress syndrome; DIC, disseminated intravascular coagulation; COPD, chronic obstructive pulmonary diseases.

### Laboratory findings

Notable disparities (*p* < 0.05) were detected in several laboratory parameters between the severe and non-severe groups. The severe group demonstrated a significantly reduced lymphocyte percentage (9.02% ± 6.07% vs. 13.42% ± 6.89%), an elevated neutrophil percentage (85.65% ± 8.81% vs. 79.55% ± 8.56%), and a diminished monocyte percentage (4.59% ± 3.02% vs. 6.55% ± 2.74%).

Concerning liver function and nutritional indicators, total bilirubin (TBil: 15.47 ± 9.91 vs. 11.55 ± 4.52 μmol/L) and direct bilirubin (DBil: 7.2 vs. 5.2 μmol/L) were increased in the severe group, whereas albumin (ALB: 31.46 ± 6.11 vs. 35.35 ± 5.65 g/L) was significantly lower. Serum sodium (Na: 136.74 ± 3.75 vs. 134.62 ± 3.92 mmol/L) was also higher in the severe group.

Markers of tissue injury, inflammation, and coagulopathy were significantly elevated in severe cases, including lactate dehydrogenase (LDH: 415.10 ± 201.35 vs. 306.20 ± 152.64 U/L), procalcitonin (PCT: 1.4 vs. 0.2 μg/L), D-dimer (2.2 vs. 0.9 mg/L), and blood urea nitrogen (BUN: 5.7 vs. 4.8 mmol/L).

Conversely, no statistically significant differences (*p* ≥ 0.05) were identified in the following parameters: white blood cell count (WBC), hemoglobin (HGB), platelet count (PLT), globulin (GLB), albumin/globulin ratio (A/G), alanine aminotransferase (ALT), aspartate aminotransferase (AST), creatinine (Cr), potassium (K), chloride (Cl), creatine kinase (CK), erythrocyte sedimentation rate (ESR), C-reactive protein (CRP), and interleukin-6 (IL-6) (Table 3).

**Table 3:** Laboratory examination on admission of patients with CPP.

Characteristics	CPP (*n* = 74)	Non severe CPP (*n* = 53)	Severe CPP (*n* = 21)	*p*-value
WBC (10^9^ L)	7.72 ± 3.53	7.37 ± 2.88	8.61 ± 4.77	0.173
L (%)	12.17 ± 6.92	13.42 ± 6.89	9.02 ± 6.07	0.013
N (%)	81.28 ± 9.01	79.55 ± 8.56	85.65 ± 8.81	0.008
M (%)	5.99 ± 2.94	6.55 ± 2.74	4.59 ± 3.02	0.009
HGB (g/L)	115.72 ± 17.89	116.94 ± 17.95	112.62 ± 17.79	0.352
PLT (10^9^ L)	210.19 ± 85.13	214.47 ± 79.20	199.38 ± 99.86	0.496
CRP (mg/L)	146.36 ± 65.12	141.20 ± 64.49	159.39 ± 66.44	0.282
ALB (g/L)	34.24 ± 6.00	35.35 ± 5.65	31.46 ± 6.11	0.011
GLB (g/L)	28.96 ± 8.27	28.47 ± 5.13	30.19 ± 13.39	0.424
A/G	1.26 ± 0.33	1.28 ± 0.30	1.20 ± 0.40	0.348
ALT (U/L)	44.5 (25.0, 73.0)	44.0 (26.0, 73.0)	45.0 (19.0, 71.0)	0.569
AST (U/L)	53.5 (33.0, 93.5)	46.0 (32.0, 74.0)	72.0 (45.0, 100.0)	0.215
TBil (umol/L)	12.66 ± 6.68	11.55 ± 4.52	15.47 ± 9.91	0.022
DBil (umol/L)	5.8 (4.0, 8.5)	5.2 (3.8, 7.8)	7.2 (4.3, 10.0)	0.041
BUN (mmol/L)	5.3 (4.0, 6.6)	4.8 (3.9, 6.0)	5.7 (4.6, 8.9)	0.037
Creatinie (umol/L)	80.95 ± 42.72	75.74 ± 26.83	94.10 ± 67.35	0.096
K (mmol/L)	3.64 ± 0.51	3.63 ± 0.50	3.69 ± 0.53	0.648
Na (mmol/L)	135.22 ± 3.97	134.62 ± 3.92	136.74 ± 3.75	0.037
Cl (mmol/L)	100.59 ± 4.67	99.99 ± 4.84	102.10 ± 3.91	0.080
LDH (U/L)	337.96 ± 174.11	306.20 ± 152.64	415.10 ± 201.35	0.015
CK (U/L)	98.0 (53.0, 292.8)	91.0 (54.0, 251.0)	116.0 (53.0, 694.0)	0.414
ESR (mm/h)	93.45 ± 32.69	92.92 ± 31.68	95.00 ± 36.80	0.845
PCT (ug/L)	0.3 (0.1, 0.9)	0.2 (0.1, 0.5)	1.4 (0.3, 3.9)	<0.001
IL-6 (pg/mL)	33.5 (16.4, 88.8)	29.9 (16.3, 61.7)	88.1 (17.4, 176.4)	0.069
D-dimer (mg/L)	1.1 (0.7, 2.2)	0.9 (0.6, 1.5)	2.2 (1.1, 3.8)	<0.001
FIB (g/L)	6.79 ± 1.80	6.90 ± 1.88	6.54 ± 1.63	0.453

CPP, *Chlamydia psittaci* pneumonia; WBC, white blood cell count; L, lymphocyte; N, neutrophil; M, monocyte; HGB, haemoglobin; PLT, platelet count; CRP, C-reactive protein; ALB, albumin; GLB, globulin; ALT, alanine transaminase; AST, aspartate transaminase; TBil, total bilirubin; DBil, direct bilirubin; BUN, blood urea nitrogen; LDH, lactate dehydrogenase; CK, creatine kinase; ESR, erythrocyte sedimentation rate; PCT, procalcitonin; IL-6, interleukin-6; FIB, fibrinoge.

### Chest CT characteristics

**Figure 2:**
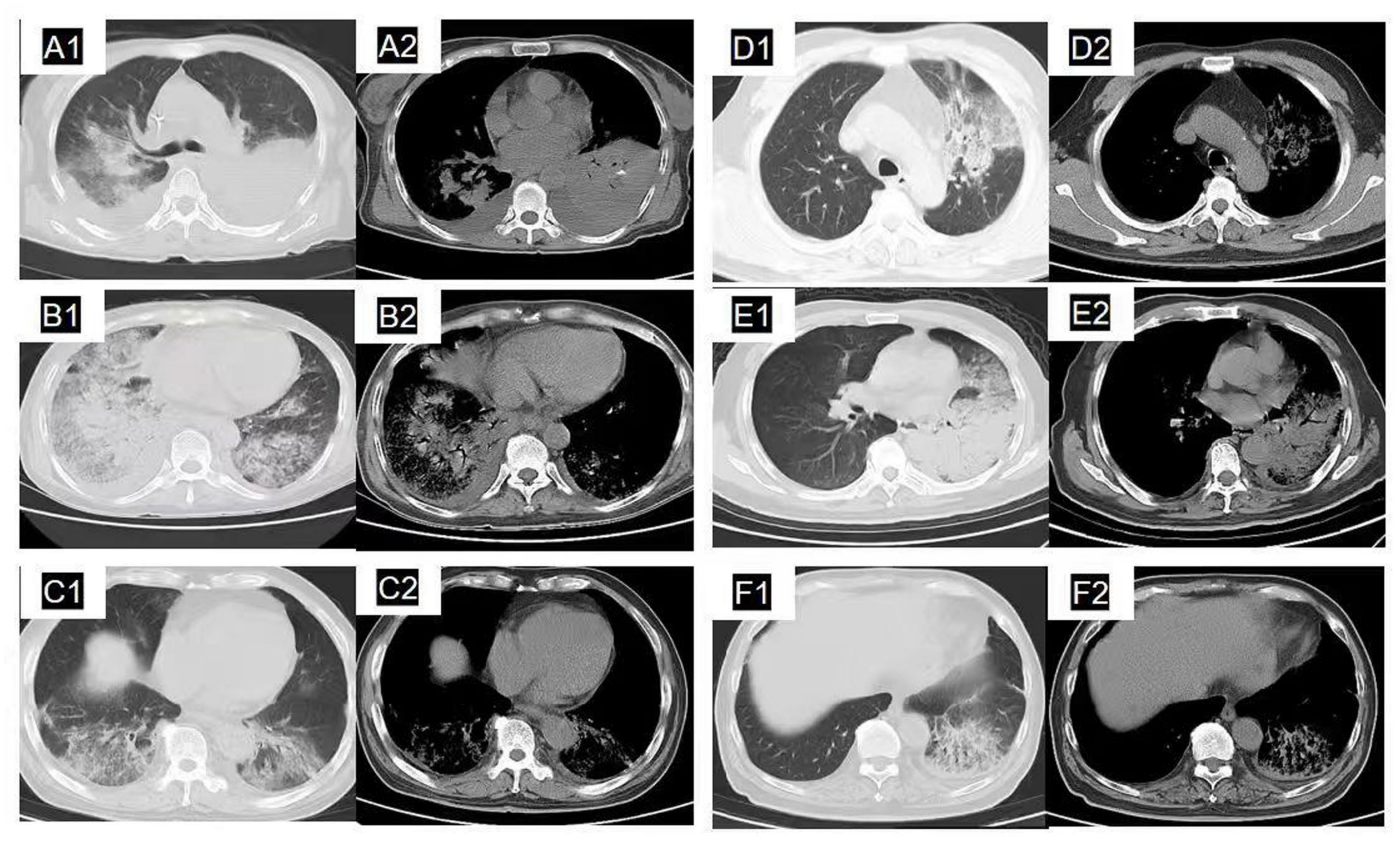
Pulmonary computed tomography findings in six cases of severe CPP. **(A1, A2)** Lung window imaging demonstrates small patchy ground-glass opacities and patchy high-density shadows in the right upper lobe, characterized by indistinct margins. In the corresponding mediastinal window, patchy consolidation is observed in the right upper lobe, accompanied by minimal bilateral pleural effusion and left lung atelectasis. **(B1, B2)** The lung window exhibits extensive patchy ground-glass opacities accompanied by patchy and small-patchy high-density shadows in the right middle and lower lobes, with ill-defined margins. Additionally, small patchy ground-glass opacities are observed in the left lower lobe. The mediastinal window shows patchy and small-patchy consolidation in the right middle and lower lobes, with visible air bronchograms. There is minimal right pleural effusion and left pleural thickening. **(C1, C2)** The lung window demonstrates extensive patchy ground-glass opacities featuring ill-defined margins and air bronchograms in both lower lobes. The mediastinal window discloses scattered patchy consolidations in both lower lobes and bilateral pleural thickening. **(D1, D2)** On the lung window, extensive, ill-defined, patchy ground-glass opacities are observed in the left upper lobe. In the mediastinal window, multiple patchy consolidations are present in the left upper lobe, accompanied by mild thickening of the right pleura. **(E1, E2)** On the lung window, large patchy regions of high-density and ground-glass opacities are observed in the left lingular segment and lower lobe, featuring indistinct margins. In the mediastinal view, extensive consolidation is evident in the left lung, accompanied by prominent air bronchograms and thickening of the left pleura. **(F1, F2)** The lung window reveals extensive patchy ground-glass opacities in the left lower lobe, characterized by ill-defined margins and the presence of air bronchograms. The mediastinal view shows linear and patchy opacities in the left lower lobe, accompanied by air bronchograms and thickening of the left pleura.

Chest CT imaging demonstrated that the distribution of pulmonary lesions was as follows: 36.5% of the lesions were located in the right lung, 32.4% in the left lung, and 31.1% were bilateral. The primary imaging manifestations included ground-glass opacities surrounding lobar consolidation, accompanied by interstitial infiltration, which was consistent with previous research (16). In 43.2% of the patients, the lesions were restricted to a single lung lobe, while in 56.8% of the patients, two or more lobes were involved, predominantly in the lower lungs. Pleural effusion was present in 54.1% (40/74) of all cases, a finding consistent with previous studies (17). However, the incidence of pleural effusion was significantly higher in the severe group compared to the non-severe group (76.2% vs. 45.3%, *p* = 0.016). Bilateral lung involvement was also significantly more prevalent in the severe group (52.4% vs. 22.6%, *p* = 0.013), indicating more extensive pulmonary pathology. Although there was no significant difference in the overall proportion of unilateral lesions (left or right) between the two groups, the proportion of unilateral right-sided lesions was notably lower in the severe group (19.0% vs. 43.4%, *p* = 0.063), suggesting a tendency towards bilateral or multifocal involvement in severe cases. Similarly, although the difference in the distribution between single-lobar and multi-lobar lesions was not statistically significant (*p* = 0.109), the frequency of multi-lobar lesions was higher in the severe group (71.4% vs. 50.9%), further corroborating the pattern of more diffuse pulmonary involvement in severe psittacosis (Table 4 and Figure 2).

**Table 4:** Radiological manifestations of patients with CPP.

Characteristics	CPP (*n* = 74)	Non-severe CPP (*n* = 53)	Severe CPP (*n* = 21)	*p*-value
Pleural effusions	40 (54.1%)	24 (45.3%)	16 (76.2%)	0.016
Scope of lesions, *n*				
Unilateral, left	24 (32.4%)	18 (34.0%)	6 (28.6%)	0.655
Unilateral, right	27 (36.5%)	23 (43.4%)	4 (19.0%)	0.063
Bilateral	23 (31.1%)	12 (22.6%)	11 (52.4)	0.013
Single lobe	32 (43.2%)	26 (49.1%)	6 (28.6%)	0.109
Multiple lobes	42 (56.8%)	27 (50.9%)	15 (71.4%)	0.109

### Predictors for severe CPP

In univariate analysis, multiple indicators exhibited significant correlations with disease severity (*p* < 0.05). D-dimer (OR = 2.113, 95% CI: 1.292–3.455, *p* = 0.003), PCT (OR = 1.594, 95% CI: 1.067–2.380, *p* = 0.023), and LDH (OR = 1.003, 95% CI: 1.000–1.007, *p* = 0.025) were significantly associated with severe psittacosis. Other indicators, including neutrophil percentage, lymphocyte percentage, monocyte percentage, alanine aminotransferase, total bilirubin, direct bilirubin, and sodium, also demonstrated correlations but necessitate further validation (Table 5). Univariate binary logistic regression analysis was conducted on the biochemical indicators at admission, indicating that multiple indicators were significantly correlated with disease severity (*p* < 0.05). L (%) (OR = 0.865, 95% CI: 0.769–0.974, *p* = 0.017), N (%) (OR = 1.105, 95% CI: 1.020–1.196, *p* = 0.014), M (%) (OR = 0.782, 95% CI: 0.635–0.964, *p* = 0.021), ALB (OR = 0.897, 95% CI: 0.818–0.984, p = 0.021), BUN (OR = 1.239, 95% CI: 1.015–1.512, *p* = 0.035), Na (OR = 1.176, 95% CI: 1.019–1.356, *p* = 0.026), LDH (OR = 1.003, 95% CI: 1.000–1.006, *p* = 0.033), PCT (OR = 1.563, 95% CI: 1.050–2.325, *p* = 0.028), and D-dimer (OR = 2.037, 95% CI: 1.248–3.324, *p* = 0.004) were significantly associated with severe psittacosis (Table 5).

**Table 5:** Univariate logistic regression analysis of laboratory indicators and early warning of severe CPP.

Characteristic	*B*	S.E.	Wald	df	*p*	OR	95% CI	
							Lower	Upper
WBC	0.104	0.077	1.802	1	0.179	1.109	0.953	1.291
L (%)	−0.144	0.06	5.749	1	0.017	0.865	0.769	0.974
N (%)	0.100	0.041	5.987	1	0.014	1.105	1.020	1.196
M (%)	−0.245	0.107	5.294	1	0.021	0.782	0.635	0.964
HGB	−0.014	0.014	1.004	1	0.316	0.986	0.959	1.014
PLT	−0.002	0.003	0.364	1	0.546	0.998	0.992	1.004
CRP	0.004	0.004	0.777	1	0.378	1.004	0.996	1.012
ALB	−0.109	0.047	5.310	1	0.021	0.897	0.818	0.984
GLB	0.019	0.030	0.421	1	0.516	1.019	0.962	1.081
A/G	−0.664	0.852	0.608	1	0.436	0.515	0.097	2.736
ALT	−0.006	0.006	1.005	1	0.316	0.994	0.983	1.005
AST	0.001	0.004	0.062	1	0.804	1.001	0.993	1.009
TB	0.077	0.043	3.198	1	0.074	1.080	0.993	1.175
DB	0.139	0.072	3.714	1	0.054	1.150	0.998	1.325
BUN	0.214	0.102	4.452	1	0.035	1.239	1.015	1.512
Creatinine	0.009	0.007	1.856	1	0.173	1.009	0.996	1.022
K	0.254	0.560	0.205	1	0.650	1.289	0.430	3.865
Na	0.162	0.073	4.940	1	0.026	1.176	1.019	1.356
Cl	0.117	0.061	3.648	1	0.056	1.124	0.997	1.268
LDH	0.003	0.002	4.537	1	0.033	1.003	1.000	1.006
CK	0.001	0.001	1.180	1	0.277	1.001	1.000	1.002
ESR	0.001	0.010	0.006	1	0.940	1.001	0.981	1.021
PCT	0.446	0.203	4.844	1	0.028	1.563	1.050	2.325
IL-6	0.004	0.002	2.546	1	0.111	1.004	0.999	1.008
D-dimer	0.711	0.250	8.100	1	0.004	2.037	1.248	3.324
FIB	−0.120	0.156	0.588	1	0.443	0.887	0.653	1.205

CPP, *Chlamydia psittaci* pneumonia; WBC, white blood cell count; L, lymphocyte; N, neutrophil; M, monocyte; HGB, haemoglobin; PLT, platelet count; CRP, C-reactive protein; ALB, albumin; GLB, globulin; ALT, alanine transaminase; AST, aspartate transaminase; TBil, total bilirubin; DBil, direct bilirubin; BUN, blood urea nitrogen; LDH, lactate dehydrogenase; CK, creatine kinase; ESR, erythrocyte sedimentation rate; PCT, procalcitonin; IL-6, interleukin-6; FIB, fibrinogen.

To identify the independent factors influencing the onset of severe psittacosis, we incorporated the significant indicators from univariate logistic analysis, along with demographic features (age, sex, height, and weight), as variables into a multivariate binary logistic regression model. A step-wise regression method was utilized for analysis. The final model retained only two statistically significant independent influencing factors: L (%) and D-dimer. The protective factor was L (%) (B = − 0.207, OR = 0.813, *p* = 0.026, 95% CI = 0.677–0.976), whereas the predictor was D-dimer (B = 1.007, OR = 2.737, *p* = 0.007, 95% CI = 1.316–5.695) (Table 6).

**Table 6:** Stepwise multivariate Logistic regression for predictors of severe CPP.

Item	B	S.E	*z*	Wald *χ*^2^	*p*	OR	OR 95% CI
L (%)	−0.207	0.093	−2.226	4.957	0.026	0.813	0.677 ~ 0.976
D-dimer	1.007	0.374	2.694	7.259	0.007	2.737	1.316 ~ 5.695
Intercept	−0.266	1.039	−0.256	0.066	0.798	0.767	0.100 ~ 5.870

L, lymphocyte.

### Diagnostic performance

**Figure 3:**
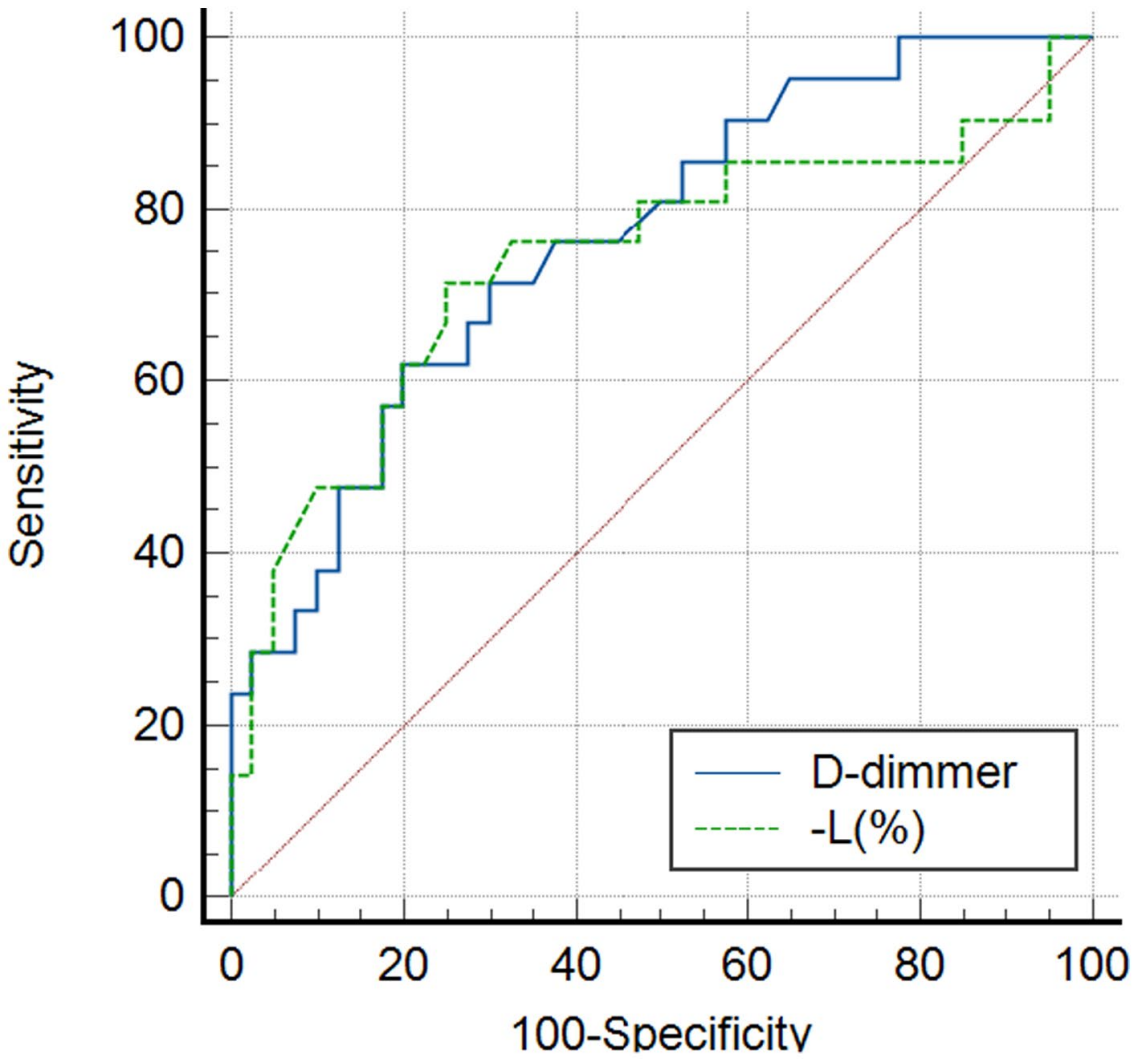
ROC curve analysis of diagnostic efficacy.

To further evaluate the predictive utility of D-dimer and L (%) for severe psittacosis, a receiver operating characteristic (ROC) curve analysis was performed (Table 7 and Figure 3). The results indicated that the area under the curve (AUC) for D-dimer was 0.765 (95% confidence interval: 0.642–0.889), which reached statistical significance (*p* = 0.001). Moreover, the AUC for the reduction of L (%) was 0.739 (95% confidence interval: 0.594–0.885) (*p* = 0.002). When the cutoff value was set at ≧1.260 mg/L for D-dimer or ≦ 9.45 for the reduction of L (%), these biomarkers exhibited high sensitivity and specificity in the prediction of severe psittacosis, implying their potential as reliable clinical auxiliary warning indices.

**Table 7:** ROC curve of LDH, PCT, D-dimer for predicting the severity of CPP.

Index	Cutoff	AUC	*p*-value	Sensitivity (%)	Specificity (%)	95%CI	
						Lower limit	Upper limit
L (%)	−9.45	0.739	0.002	71.4	75.0	0.594	0.885
D-dimer	1.260 mg/L	0.765	0.001	71.4	70.0	0.642	0.889

L, lymphocyte.

### Treatment and prognosis

The overall average duration of fever during hospitalization was 8.81 ± 4.76 days. There was no significant difference in the average duration of fever between the non-severe group (8.40 ± 4.77 days) and the severe group (9.86 ± 4.33 days) (*p* = 0.227). Tetracycline antibiotics were the primary treatment option, accounting for 70.3% of the cases. The usage of tetracycline antibiotics did not differ significantly between the two groups (*p* = 0.891). The overall utilization rate of quinolones was relatively low, at 10.8%. Notably, quinolones were not used in the severe group (0% in the severe group vs. 15.1% in the non-severe group), and this difference approached statistical significance (*p* = 0.096). The rates of switching from quinolones to tetracyclines and the application of combination antibiotic therapy were low, and there were no significant differences between the groups.

A statistically significant difference was observed in the oxygenation index (PaO_2_/FiO_2_), which was markedly lower in the severe group compared to the non-severe group (200.6 ± 52.3 vs. 280.3 ± 77.2, *p* < 0.001). Consistently, the severe group required significantly higher levels of respiratory support. Invasive mechanical ventilation (IV) was employed in 14.3% of severe cases, while it was not used in non-severe cases (*p* = 0.021). Non-invasive ventilation (NIV) was administered to 33.3% of severe patients, compared to 0% in the non-severe group (*p* < 0.001). High-flow nasal cannula oxygen therapy (HFNC) was utilized in 23.8% of severe cases, whereas it was not used in non-severe cases (*p* = 0.001). Conventional oxygen therapy was commonly used in both groups, with a utilization rate of 77.0%, and there was no significant difference in its utilization between the two groups (*p* = 0.364).

Regarding clinical outcomes, the overall mortality rate was 5.4%. The mortality rate was significantly higher in the severe group (19% in the severe group vs. 0% in the non-severe group, *p* = 0.005) (Table 8).

**Table 8:** Treatment and outcomes of patients with CPP.

Characteristic	CPP (*n* = 74)	Non-severe CPP (*n* = 53)	Severe CPP (*n* = 21)	*p*-value
Fever duration	8.81 ± 4.76	8.40 ± 4.77	9.86 ± 4.33	0.227
PaO_2_/FiO_2_	254.9 ± 79.2	280.3 ± 77.2	200.6 ± 52.3	<0.001
Antimicrobial therapy				
Tetracyclin	52 (70.3%)	37 (69.8%)	15 (71.4%)	0.891
Quinolones	8 (10.8%)	8 (15.1%)	0 (0)	0.096
Quinolones to tetracyclin	12 (16.2%)	7 (13.2%)	5 (23.8%)	0.303
Combination therapy	2 (2.7%)	1 (1.9%)	1 (4.8%)	0.490
Respiratory support, *n*				
IV	3 (4.1%)	0 (0)	3 (14.3%)	0.021
NIV	7 (9.5%)	0 (0)	7 (33.3%)	<0.001
Oxygen therapy	57 (77.0%)	39 (73.6)	18 (85.7%)	0.364
HFNC	5 (6.8%)	0 (0)	5 (23.8%)	0.001
Outcomes				
Death	4 (5.4%)	0 (0)	4 (19%)	0.005

IV, invasive ventilation; NIV, non-invasive ventilation; HFNC, high-flow nasal cannula.

## Discussion

In the context of the extensive implementation of next-generation sequencing (NGS) technology, CPP has attracted growing clinical concern. Previous research suggests that CPP accounts for roughly 6.8% of community-acquired pneumonia cases (4), and severe pneumonia constitutes up to 8% of these cases (18). Humans are mainly infected with this disease by inhaling aerosols from the respiratory secretions or dried feces of infected birds and poultry (19).

This retrospective investigation analyzed 74 patients diagnosed with CPP via NGS, concentrating on the predictors and clinical manifestations of severe illness. In line with previous literature, patients generally presented with a history of avian/poultry exposure, high-grade fever, cough, expectoration, fatigue, dizziness, and myalgia. Laboratory results commonly showed normal white blood cell counts, along with mild elevations in lactate dehydrogenase (LDH), C-reactive protein (CRP), and erythrocyte sedimentation rate (ESR), accompanied by elevated D-dimer levels and coagulation disorders (20, 21).

Previously, multivariate regression analysis identified D-dimer as an independent predictor for severe disease (22, 23). As a by-product of fibrin degradation, elevated D-dimer levels signify activation of the coagulation system and hyperfibrinolysis. Severe pneumonia frequently involves dysregulation of the immunocoagulation cascade, potentially leading to microthrombosis or disseminated intravascular coagulation (DIC). In this study, D-dimer levels were significantly elevated in the severe group. Furthermore, receiver operating characteristic (ROC) curve analysis demonstrated its robust predictive capacity (AUC = 0.765), underscoring the clinical importance of early dynamic monitoring for identifying high-risk patients.

Likewise, the lymphocyte percentage (L%) was recognized as a protective factor for severe psittacosis, suggesting that lower L% levels are correlated with a higher probability of developing severe disease. Specifically, for every 1-unit reduction in L%, the odds of developing severe psittacosis increase by approximately 18.7%. These results imply that elevated D-dimer and significantly low L% levels act as crucial warning signs for disease progression.

In terms of imaging characteristics, the findings were consistent with previous reports, commonly presenting lobar consolidation surrounded by ground-glass opacities and interstitial abnormalities (16). Pleural effusion was present in 60% of cases (17). This study found a comparable overall pleural effusion rate of 54.1%, yet with significantly higher proportions in the severe group (76.2%) and a greater incidence of bilateral lung involvement (52.4%). This pattern implies that extensive pleural effusion and bilateral lesions on imaging should prompt clinicians to be aware of a higher risk of severe disease, presumably associated with increased capillary permeability and aggravated inflammatory exudation in severe infections.

Although the clinical manifestations of CPP are intricate and non-specific, timely diagnosis and management are of paramount importance to avert unfavorable outcomes. Next-generation sequencing (NGS) technology presents remarkable advantages in pathogen detection, facilitating early targeted therapy and curbing inappropriate antibiotic utilization (6). A multitude of studies have successfully identified *C. psittaci* in bronchoalveolar lavage fluid (BALF) or lung tissue through metagenomic next-generation sequencing (mNGS), wherein the quantity of pathogen-specific sequences can serve as an indicator of active infection (24). The detection of *C. psittaci* DNA by this approach should arouse a high degree of suspicion for psittacosis. In this study, one patient with a low count of blood mNGS sequences still manifested clinical symptoms and treatment responses consistent with CPP, which is in line with previous research (7). Targeted next-generation sequencing (tNGS) also showcases high sensitivity, rapid turnaround time, and cost-effectiveness for lower respiratory tract pathogens, rendering it another valuable instrument for clinical diagnosis (25). However, reports on its application in confirming psittacosis remain scarce.

Previous research has confirmed the high effectiveness of quinolones and tetracyclines in the treatment of community acquired pneumonia, characterized by rapid defervescence and high recovery rates (6). The present study validates these findings: 70.3% of patients were administered tetracycline monotherapy, 10.8% received quinolone monotherapy, and combination or sequential therapy was applied in a small number of cases. The overall mortality rate was 5.4%, mainly observed in severe cases accompanied by complications, mixed infections, or significant comorbidities. Significantly, 33.3% of severe patients needed invasive mechanical ventilation, and their hospital stays were notably extended, which underscores the considerable healthcare burden and life-threatening nature of severe CPP. Although all patients eventually received targeted antimicrobial agents, early identification of high-risk individuals and aggressive respiratory support are crucial for enhancing treatment outcomes.

Our analysis of predictive factors confirmed that elevated D-dimer levels, which are significantly higher in severe cases, constitute a reliable indicator of disease severity. Binary logistic regression and ROC curve analysis demonstrated that a D-dimer cutoff value of ≥1.260 mg/L and a lymphocyte percentage reduction cutoff value of ≤9.45% both exhibited significant diagnostic efficacy. These thresholds demonstrated sensitivities of 71.4 and 71.4%, and specificities of 70.0 and 75.0%, respectively, for predicting severe psittacosis. Dynamic monitoring of these biomarkers can facilitate the early identification of high-risk patients. Nevertheless, the single-center design and limited sample size of this study necessitate further prospective, multicenter investigations involving larger cohorts to validate the generalizability of these biomarker thresholds.

## Conclusion

In summary, CPP manifests with an acute onset, and severe cases are in a critical condition. Bilateral pulmonary involvement, pleural effusion, and significant elevations in D-dimer and LDH levels serve as crucial indicators for discerning severe cases. D-dimer can be considered a reliable biomarker for predicting the severity of the disease. In clinical practice, heightened vigilance should be exercised for patients with a history of avian exposure exhibiting these characteristics. Early identification of high-risk patients is of paramount importance, and confirmed cases should be promptly administered a full-course of tetracycline therapy. Timely respiratory support should be provided in accordance with the progression of the disease to halt deterioration and improve the prognosis.

This research exhibits several limitations: (1) It is a single-center study with a comparatively restricted sample size; (2) The inclusion of only cases confirmed by next-generation sequencing (NGS) might introduce selection bias, as a multitude of clinically compatible yet undiagnosed cases were excluded; (3) The severity of pneumonia was not assessed using standardized scoring systems such as the Pneumonia Severity Index (PSI) or the CURB-65 score. Future investigations should focus on multi-center collaborations and the incorporation of validated severity scores to improve the early recognition and management of psittacosis, thereby ultimately decreasing the incidence and mortality of severe cases.

## Data Availability

The raw data supporting the conclusions of this article will be made available by the authors, without undue reservation.
