# Supplementary material for: Clinical features and prognostic factors of Chlamydia psittaci pneumonia: a retrospective study
Source: Front Med (Lausanne). 2026 Apr 2;13:1804156. doi: 10.3389/fmed.2026.1804156 (PMC13083060; doi:10.3389/fmed.2026.1804156)
Supplement: Supplementary file 1 [file Table_1.docx]

Supplementary Appendix: Definitions and Diagnostic Criteria for Complications

1. Respiratory Failure

The diagnosis of respiratory failure was established based on arterial blood gas analysis. It was defined as a partial pressure of arterial oxygen (PaO_2_) below 60 mmHg while breathing room air at sea level, with or without a partial pressure of arterial carbon dioxide (PaCO_2_) exceeding 50 mmHg, the latter typically accompanied by respiratory acidosis (pH < 7.35) ^[1]^.

2. Sepsis

Sepsis was conceptualized as life-threatening organ dysfunction resulting from a dysregulated host response to infection, in accordance with the Sepsis-3 consensus^[2]^. Operationally, it was quantified by an acute increase of 2 points or more in the Sequential Organ Failure Assessment (SOFA) score attributable to infection. For bedside screening in non-intensive care settings, a positive quick SOFA (qSOFA) score, defined by the presence of at least two of the following criteria—tachypnea (respiratory rate ≥ 22 breaths/min), altered mentation (Glasgow Coma Scale < 15), or hypotension (systolic blood pressure ≤ 100 mmHg)—was utilized to identify patients at high risk.

3. Acute Respiratory Distress Syndrome (ARDS)

The identification of ARDS was conducted in accordance with the Berlin Definition^[3]^, which required the fulfillment of four key elements: (1) onset within one week of a known clinical insult or new/worsening respiratory symptoms; (2) bilateral opacities on chest imaging (radiograph or computed tomography) not fully explained by effusions, lobar collapse, or nodules; (3) respiratory failure not entirely attributable to cardiac failure or fluid overload, necessitating objective evaluation (e.g., echocardiography) to exclude hydrostatic edema; and (4) the severity of hypoxemia, graded by the PaO_2_/FiO_2_ ratio with a minimum positive end-expiratory pressure (PEEP) of 5 cm H2O, as mild (201-300 mmHg), moderate (101-200 mmHg), or severe (≤100 mmHg) .

4. Acute Kidney Injury

According to the Kidney Disease: Improving Global Outcomes (KDIGO) criteria^[4]^, acute kidney injury was defined by the occurrence of any of the following: an increase in serum creatinine by ≥ 0.3 mg/dL (≥ 26.5 µmol/L) within 48 hours; an increase in serum creatinine to ≥1.5 times baseline, known or presumed to have occurred within the prior 7 days; or a urine volume of less than 0.5 mL/kg/h sustained for 6 hours.

5. Cardiac Failure

A diagnosis of acute cardiac failure required an integrated assessment of clinical presentation and objective findings ^[5]^. Essential indicators included classic symptoms (dyspnea, orthopnea, paroxysmal nocturnal dyspnea, fatigue) and physical signs (pulmonary crackles/rales, bilateral pedal edema, elevated jugular venous pressure). Objective confirmation was obtained via elevated natriuretic peptide levels (BNP > 100 pg/mL or NT-proBNP > 300 ng/L) and/or echocardiographic evidence of structural or functional cardiac abnormalities (e.g., reduced left ventricular ejection fraction).

6. Anemia

Anemia was defined according to the thresholds established by the World Health Organization (WHO)^[6]^. The condition was identified by a hemoglobin concentration below 13.0 g/dL (130 g/L) in adult males, below 12.0 g/dL (120 g/L) in non-pregnant adult females, and below 11.0 g/dL (110 g/L) in pregnant females .

7. Disseminated Intravascular Coagulation (DIC)

The diagnosis of overt disseminated intravascular coagulation (DIC) was established using the International Society on Thrombosis and Haemostasis (ISTH) diagnostic scoring system [7]. A cumulative score of ≥5 was required for diagnosis, calculated as the sum of points assigned to four routine laboratory parameters: platelet count (0 points for ≥100×10⁹/L, 1 for 50–100, 2 for <50), fibrin-related markers (e.g., D-dimer; 0 for no elevation, 2 for moderate, 3 for marked elevation), prothrombin time prolongation (0 for <3 seconds above control, 1 for 3–6 seconds, 2 for >6 seconds), and fibrinogen concentration (0 for ≥1.0 g/L, 1 for <1.0 g/L). A score of ≥5 indicates overt DIC; scores below 5 suggest non-overt DIC, which was not applied in the present analysis

References

1. Schwartzstein RM, Adams L. Respiratory failure. In: Loscalzo J, Fauci A, Kasper D, et al., eds. Harrison's Principles of Internal Medicine. 21st ed. New York, NY: McGraw Hill; 2022:1945-1953.
2. Singer M, Deutschman CS, Seymour CW, et al. The Third International Consensus Definitions for Sepsis and Septic Shock (Sepsis-3). JAMA. 2016;315(8):801-810. doi:10.1001/jama.2016.0287
3. ARDS Definition Task Force, Ranieri VM, Rubenfeld GD, Thompson BT, et al. Acute respiratory distress syndrome: the Berlin Definition. JAMA. 2012;307(23):2526-2533. doi:10.1001/jama.2012.5669
4. Kidney Disease: Improving Global Outcomes (KDIGO) Acute Kidney Injury Work Group. KDIGO Clinical Practice Guideline for Acute Kidney Injury. Kidney Int Suppl. 2012;2:1-138.
5. McDonagh TA, Metra M, Adamo M, et al. 2021 ESC Guidelines for the diagnosis and treatment of acute and chronic heart failure. Eur Heart J. 2021;42(36):3599-3726. doi:10.1093/eurheartj/ehab368
6. World Health Organization. Guideline on haemoglobin cutoffs to define anaemia in individuals and populations. Geneva: World Health Organization; 2024. Available from: <https://www.who.int/publications/i/item/9789240088542>
7. Wada, H., Matsumoto, T. & Yamashita, Y. Diagnosis and treatment of disseminated intravascular coagulation (DIC) according to four DIC guidelines. *J Intensive Care* **2**, 15 (2014). <https://doi.org/10.1186/2052-0492-2-15>
